# Effects of nitroglycerin versus labetalol on peripheral perfusion during deliberate hypotension for sinus endoscopic surgery: a randomized, controlled, double-blinded trial

**DOI:** 10.1186/s12871-020-01006-w

**Published:** 2020-04-17

**Authors:** Marwa Zayed, Heba Nassar, Ahmed Hasanin, Amany H. Saleh, Passaint Hassan, Dalia Saad, Sahar Mahmoud, Ghada Abo Bakr, Eman Fouad, Norhan Saleh, Maha Ismail, Hani El-Hadi

**Affiliations:** 1 Department of Anesthesia and Critical Care Medicine, Cairo University, Cairo, Egypt; 2 Department of Anesthesia and Critical Care Medicine, Faculty of Medicine, Cairo University, 01 elsarayah street, Elmanyal, Cairo, 11559 Egypt

**Keywords:** Deliberate hypotension, Nitroglycerin, Labetalol, Peripheral perfusion, Lactate

## Abstract

**Background:**

Deliberate hypotension is used to provide a bloodless field during functional endoscopic sinus surgery; however, the impact of controlled hypotension during anesthesia on peripheral tissue perfusion has not been extensively evaluated. The aim of this study was to compare the impact of nitroglycerin- versus labetalol-induced hypotension on peripheral perfusion.

**Methods:**

The present randomized, double-blinded, controlled trial included adult patients undergoing endoscopic sinus surgery. Patients were allocated to one of two groups according to the drug received for induction of deliberate hypotension: nitroglycerin (*n* = 20) or labetalol (*n* = 20). Mean arterial pressure was maintained at 55–65 mmHg in both groups. Both study groups were compared according to pulse oximeter-derived peripheral perfusion index (primary outcome), serum lactate level, mean arterial pressure, heart rate, surgical field score, and intraoperative blood loss.

**Results:**

Forty patients were included in the final analysis. The nitroglycerin group exhibited a higher peripheral perfusion index at nearly all records (*p* < 0.0001) and lower postoperative serum lactate levels (1.3 ± 0.2 mmol/L vs. 1.7 ± 0.4 mmol/L; *p* = 0.001) than the labetalol group. The peripheral perfusion index was higher in the nitroglycerin group than at baseline at most intraoperative readings. The median surgical field score was modestly lower in the labetalol group than in the nitroglycerin group in the first 20 min (2 [interquartile range (IQR) 2–2.5] versus 1.5 [IQR 1–2]; *p* = 0.001). Both groups demonstrated comparable and acceptable surgical field scores in all subsequent readings.

**Conclusion:**

Nitroglycerin-induced deliberate hypotension was accompanied by higher peripheral perfusion index and lower serum lactate levels than labetalol-induced deliberate hypotension during sinus endoscopic surgery.

**Trial registration:**

The study was registered at clinicaltrials registry system with trial number: NCT03809065. Registered at 19 January 2019. This study adheres to CONSORT guidelines.

## Background

Controlled hypotension is commonly used during functional endoscopic sinus surgery (FESS) to ensure a bloodless operative field. The main risk in controlled hypotension is impaired peripheral perfusion and subsequent ischemia of vital organs [1, 2]. A target mean arterial pressure (MAP) of 50–65 mmHg has been considered sufficient to achieve acceptable surgical conditions [1]. However, the use of blood pressure as the only hemodynamic target is not sufficient to ensure proper oxygen delivery to different organs, which is the main goal of cardiac output. Because monitoring of vital organ perfusion requires either sophisticated or invasive equipment, the use of deliberate hypotension has been limited by the fear of unmonitored ischemia of vital organs. Monitoring of blood flow in non-vital organs (such as the skin) has been considered to be a good alternative for assessment of the impact of deliberate hypotension on tissue perfusion. Peripheral perfusion of non-vital organs is usually impaired earlier than vital organs [3]; therefore, evaluation of perfusion of non-vital organs is considered to be an adequate measure of patient safety during anesthesia [4]. Furthermore, the development of simple non-invasive measures, such as pulse oximetry-derived peripheral perfusion index (PPI), has provided effective and easy measures for tissue perfusion [3]. Recently, PPI has been reported to be a useful hemodynamic measure during anesthesia [4]. Thus, we hypothesized that monitoring PPI would be a useful tool for evaluating the safety of deliberate hypotension. Serum lactate is another frequently used marker of global tissue perfusion in the operating room as well as the intensive care unit [3].

Nitroglycerin and labetalol are commonly used drugs for controlled hypotension during anesthesia. Both drugs are also used for acute management of emergent hypertension in emergency departments. Nitroglycerin is a direct vasodilator and its main action is through venodilation. Labetalol combines an α1 blocking effect and beta-adrenergic blocking activity to reduce systemic vascular resistance, with little effect on cardiac output [5, 6]. Both drugs were previously compared during controlled hypotension with regard to surgical field quality and blood loss [6–9]. To date, however, no study has compared the effects of both drugs on peripheral perfusion.

The aim of this study, therefore, was to compare the impact of nitroglycerin and labetalol on peripheral perfusion when used for induction of deliberate hypotension during FESS. We evaluated peripheral perfusion using PPI and serum lactate levels as markers of peripheral and global tissue perfusion.

## Methods

This randomized, double-blinded, parallel study was conducted at Cairo University Hospital (Cairo, Egypt) after receiving approval (N-53-2016) from the Research Ethics Committee, Faculty of Medicine, Cairo University. Written informed consent was obtained from all participants before enrollment.

Forty adult patients 18–45 years of age, with American Society of Anesthesiologists physical status I-II and scheduled for FESS, were included. Individuals with uncontrolled hypertension, cerebrovascular disorders, coagulation disorders, cardiovascular diseases, renal impairment, liver impairment, and history of allergic reaction to any of the study medications were excluded from the study.

An online randomization program (http://www.randomizer.org) was used by a research assistant to generate random sequences. Each code was enclosed in a sealed opaque envelope. Another research assistant, who was not involved in outcome assessment, was responsible for opening the envelope and preparing the study drug. Patients were allocated before induction of anesthesia into either the nitroglycerin (*n* = 20) or labetalol (*n* = 20) group. The patient, attending anesthesiologist, and surgeon were blinded to the study group allocation.

### Anesthetic management

On arrival to the operating room, intravenous midazolam (0.05 mg/kg) and fentanyl (50 μg) were administered as premedication to all participants. Two 18-gauge peripheral intravenous catheters were secured: one for fluid administration; and the other for infusion of the study drug. Monitoring included electrocardiography, non-invasive arterial blood pressure, end-tidal carbon dioxide (CO_2_), and temperature probe. PPI was measured using a pulse oximetry probe (Masimo Radical 7; Masimo Corp., Irvine, CA, USA), which was attached to the patients’ index finger of the non-dominant hand. Invasive blood pressure was monitored using a 20-gauge radial artery catheter connected to a pressure transducer. Arterial blood gases were measured using a specialized, commercially available device (GEM Premier 3000, Bedford, MA, USA).

Anesthesia was induced using fentanyl (2 μg/kg), propofol (1.5–2 mg/kg), atracurium (0.5 mg/kg) to facilitate insertion of an appropriately sized endotracheal tube, and 1 g paracetamol for analgesia. Anesthesia was maintained using an isoflurane end-tidal concentration of 1.2% and 0.01 mg/kg atracurium every 20 min. Lactated Ringer’s solution was infused at a rate of 5 mL/kg. Mechanical ventilation was adjusted to maintain end-tidal CO_2_ at 30–35 mmHg. The head-up position was maintained at 30°, and nasal mucosa was infiltrated by the surgeon with 2 mL of 2% lignocaine with epinephrine at 1:100,000 dilution.

### Drug preparation and hemodynamic management

Nitroglycerin (Nitronal® glyceryl trinitrate, 1 mg/mL, G. Pohl-Boskamp GmbH & Co. KG, 25551 Hohenlockstedt, Germany) was diluted by adding 3 mL (3 mg) to 47 mL of 0.9% saline in a 50 mL syringe. Labetalol (Labetalol Hydrochloride Injection®, 5 mg/ml) was diluted by adding 10 mL (50 mg) to 40 mL of 0.9% saline in a 50 mL syringe. Both drugs were infused after endotracheal intubation with a starting dose of 0.5 mL/kg/h to have a starting dose of 0.5 μg/kg/min for nitroglycerin and 0.5 mg/kg/h for labetalol. The infusion was then titrated according to patient response. The rate of nitroglycerin infusion was 0.5–2 μg/kg/min, whereas the labetalol infusion rate was 0.5–2 mg/kg/h.

The infusion rate was finely adjusted to maintain invasive MAP at approximately 55–65 mmHg or until achieving the adequate surgical field (defined as surgical field score [SFS] < 3). Hypotension was defined as a decrease in MAP below the target level, which was managed by decreasing the rate of drug infusion by 50% up to transient holding of infusion. If hypotension persisted after stopping drug infusion, incremental doses of intravenous ephedrine (5 mg) were planned to be administered. Bradycardia was defined as a heart rate < 50 beats/min and was planned to be treated with intravenous atropine 0.5–1.0 mg and discontinuation of the infused drug.

The surgical field was evaluated according to SFS [10] by a surgeon, who was blinded to the study groups, and scored from 0 to 5 as follows: 0, no bleeding; 1 slight bleeding (no suctioning of blood required); 2, slight bleeding (occasional suctioning required, surgical field not threatened); 3 slight bleeding (frequent suctioning required, bleeding threatened surgical field a few seconds after suction was removed); 4, moderate bleeding (frequent suctioning required, bleeding threatened the surgical field directly after suction was removed; and 5, severe bleeding (constant suctioning required, bleeding appears faster and can be removed by suction, surgical field severely threatened and surgery not possible).

Twenty minutes before the end of the procedure, the infused drugs were gradually weaned off. At the conclusion of surgery, isoflurane was discontinued, and residual neuromuscular blockade was reversed using neostigmine 0.05 mg/kg and atropine 0.02 mg/kg.

### Outcomes

#### Primary outcome

PPI was measured at the following time points: before induction of anesthesia; 1 min after endotracheal intubation; every 5 min after initiation of study drug infusion until the end of the procedure; and every 10 min in the post-anesthesia care unit for 60 min. During data analysis, measurements were averaged every 15 min until the end of the operation; the postoperative 60 min was averaged in one point.

#### Secondary outcomes

Serum lactate concentration: Two samples were obtained at baseline and 60 min after extubation. Total intraoperative blood loss was measured from the suction device and gauze that was used to dry the surgical field. SFS was measured every 5 min intraoperatively until the end of the procedure. Each two successive measurements were averaged (every 10 min) during analysis.

Mean arterial blood pressure and heart rate were assessed at the following time points: pre-induction, 1 min after endotracheal intubation (intubation was performed 2 min after induction of anesthesia); every 5 min after initiation of study drug infusion until the end of the procedure, and every 10 min in the post-anesthesia care unit for 60 min. During data analysis, intraoperative measurements were averaged every 15 min; the postoperative 60 min was averaged in one point.

### Statistical analysis

In a pilot study involving 5 patients, an intraoperative, average PPI was reported during the first 1 h of anesthesia after nitroglycerin infusion at a mean ratio of 4.5 ± 0.8 during the same operation. A sample size that could detect 20% difference in PPI (i.e., 0.9) between the two study groups was calculated using MedCalc software version 14.10.2 (MedCalc Software bvba, Ostend, Belgium). A minimum of 34 patients (17 patients per group) was calculated to have a study power of 80% and an alpha error of 0.05. The number was increased to 40 patients (20 patients per group) to compensate for possible/projected dropouts. We chose 20% mean difference in the PPI during sample size calculation because this relative change was previously reported as an accurate index for increased cardiac output after passive leg raising [11].

SPSS version 15 (IBM Corporation, Chicago, IL, USA) for Windows (Microsoft Corporation, Redmond, WA, USA) was used for data analysis. Categorical data are expressed as frequency (percentage [%]) and analyzed using the chi-squared test. Normality of continuous data was verified using the Shapiro-Wilk test, which are expressed as mean ± standard deviation (SD) or median (interquartile range [IQR]), as appropriate. Continuous data were analyzed using the unpaired t test or the Mann-Whitney test as appropriate. Repeated measures were analyzed using analysis of variance (ANOVA) for repeated measures with post-hoc pairwise comparisons using the Bonferroni test. Differences with *P* < 0.05 were considered to be statistically significant.

## Results

**Fig. 1:**
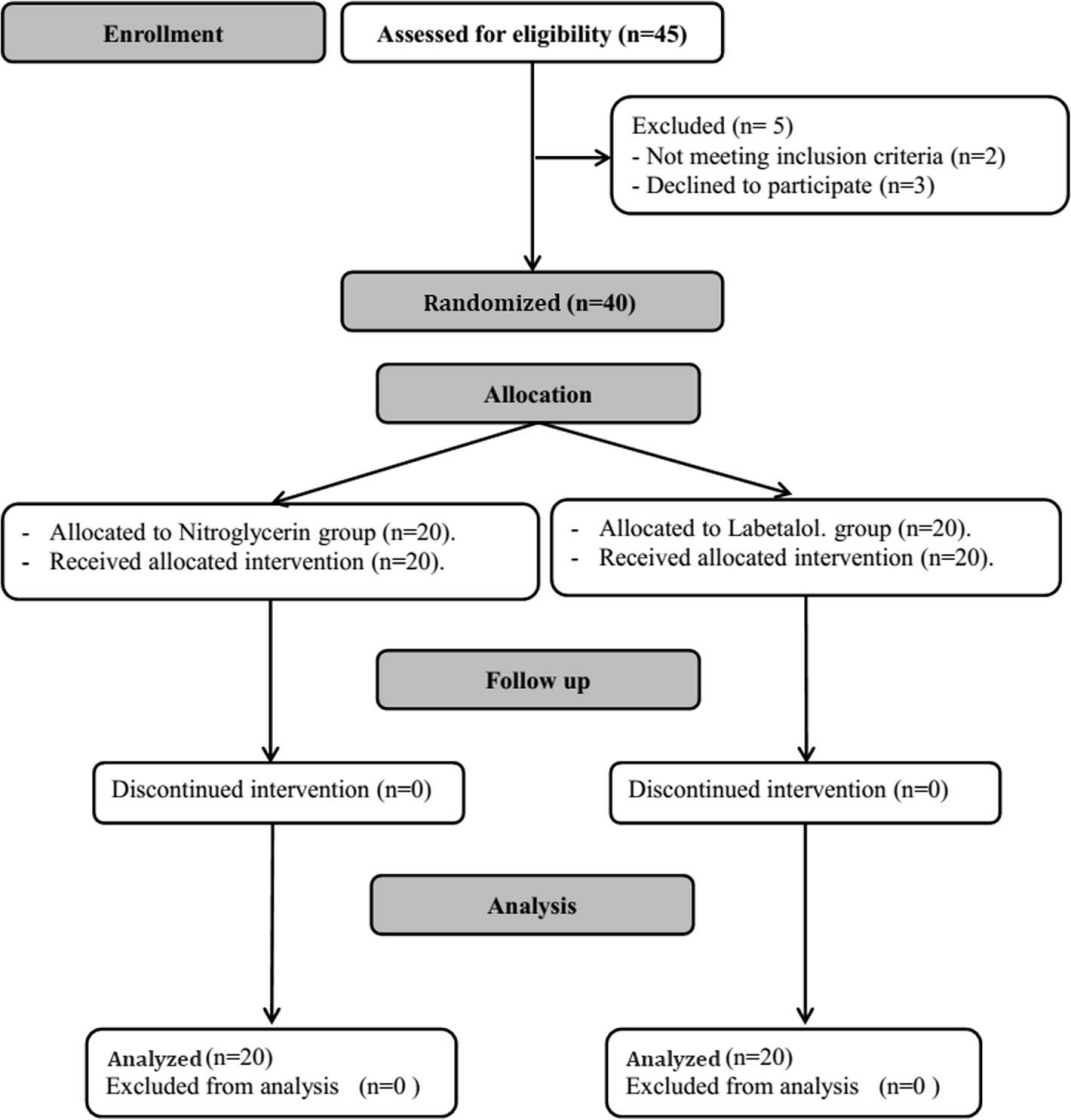
Flow diagram showing patients’ recruitment

Forty-five patients, recruited from January to May 2019, were screened for eligibility in this study. Two patients did not meet the inclusion criteria, three declined to participate, and 40 were randomly assigned to receive one of the two interventions. All patients completed the intervention and data were available for final analysis (Fig. 1). Patient characteristics and operative data were comparable between the study groups; with the exception of time to reach target SFS, which was lower in the labetalol group (Table 1). The mean nitroglycerin infusion rate and total infused dose were 1.7 ± 0.3 μg/kg/min and 13.9 ± 3.5 mg, respectively. Furthermore, the mean labetalol infusion rate and total infused dose were 0.8 ± 0.2 mg/kg/h and 98.7 ± 19.7 mg, respectively. No patient in either group required doses of rescue atropine or ephedrine.

**Table 1:** Demographic and surgical data

Data	Nitroglycerin group (*n* = 20)	Labetalol group (*n* = 20)	*P* value
Age (years)	30.1 ± 4.8	30.6 ± 5.7	0.791
Weight(Kg)	76.9 ± 9.4	77.3 ± 8.2	0.901
Gender(M/F)	12/8	11/9	0.749
ASA(I/II)	15/5	16/4	0.704
Duration of anesthesia (min)	140 ± 20.3	143.7 ± 15.9	0.520
Duration of controlled hypotension (min)	101.5 ± 10.8	104.2 ± 8.7	0.401
Time to reach target SFS (min)	11 (10–20)	5(5–15)*	0.010
Total fentanyl consumption (μg)	227.5 ± 25.5	215 ± 22.1	0.105

Data are presented as mean ± SD, frequency, or median (interquartile range). * Denotes significance compared with nitroglycerin group; *P* < 0.05. Data were analyzed using the chi-squared test, the unpaired t test and the Mann-Whitney test as appropriate

**Fig. 2:**
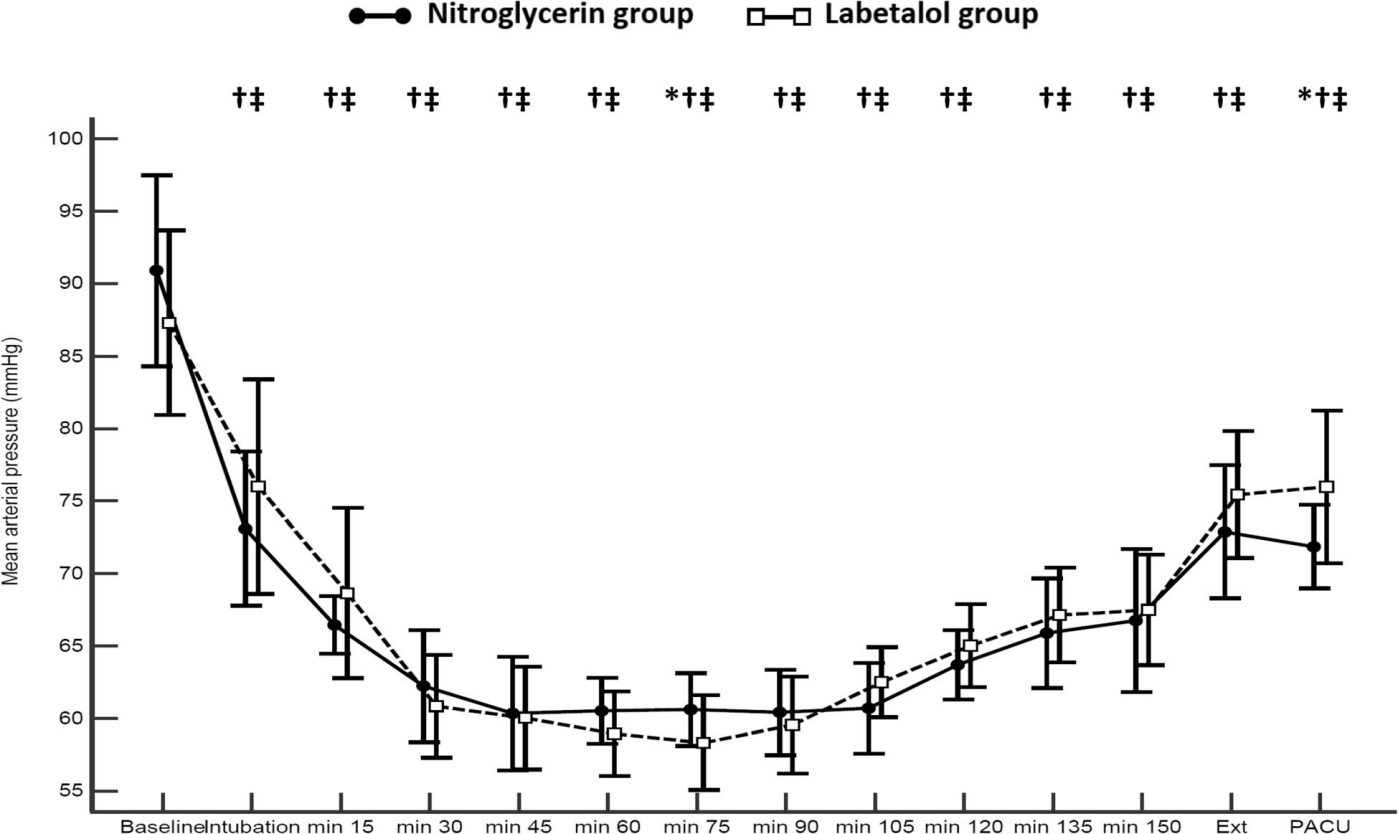
Mean arterial pressure at time points between groups. Markers are means, error bars are standard deviations. Ext: extubation, PACU: post-anesthesia care unit. * denotes significance between both groups., † denotes significance compared to the baseline reading within nitroglycerin group, ‡ denotes significance compared to the baseline reading within labetalol group

**Fig. 3:**
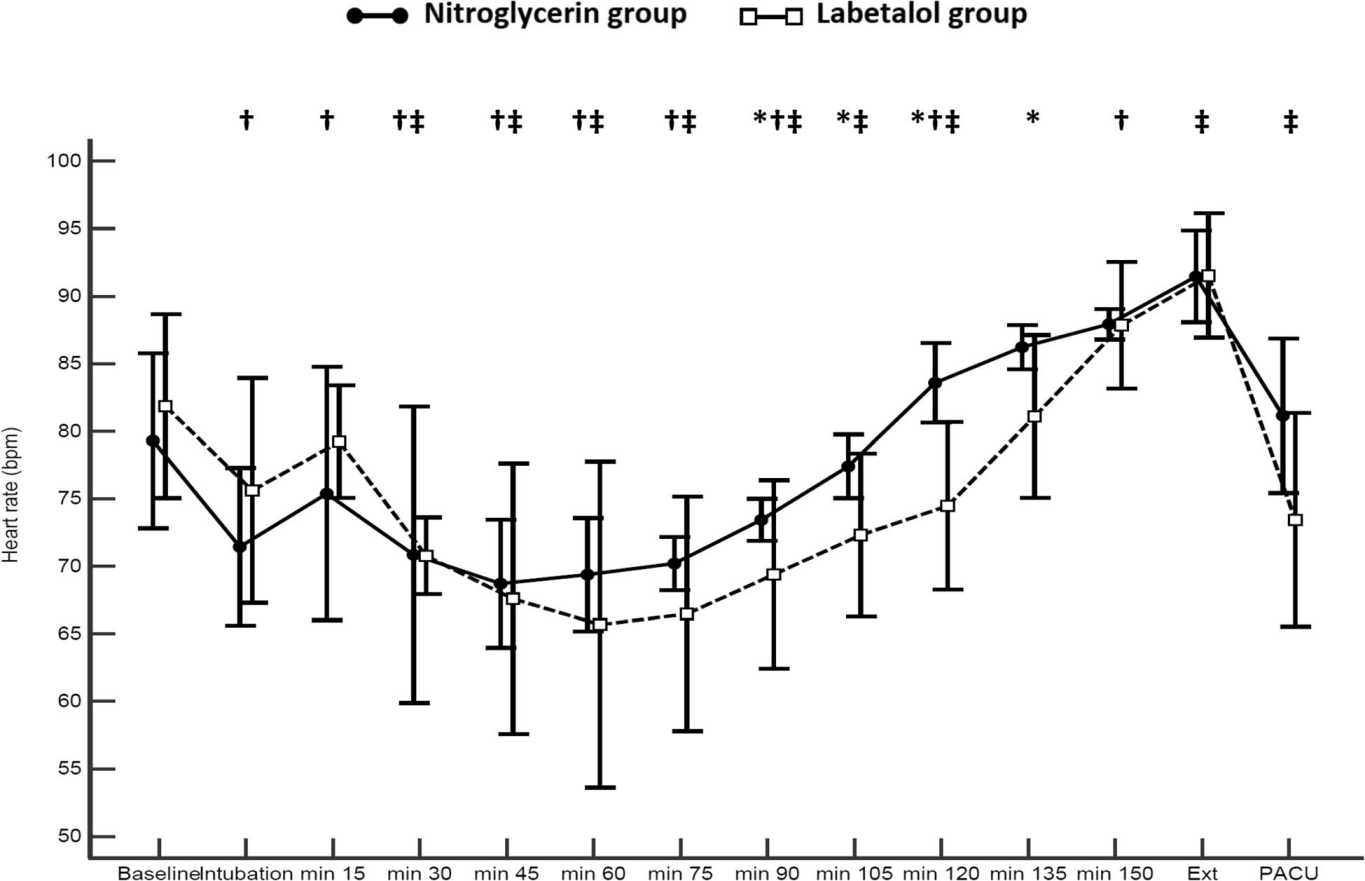
Heart rate at time points between groups. Markers are means, error bars are standard deviations. Ext: extubation, PACU: post-anesthesia care unit. * denotes significance between both groups, † denotes significance compared to the baseline reading within nitroglycerin group, ‡ denotes significance compared to the baseline reading within labetalol group

**Fig. 4:**
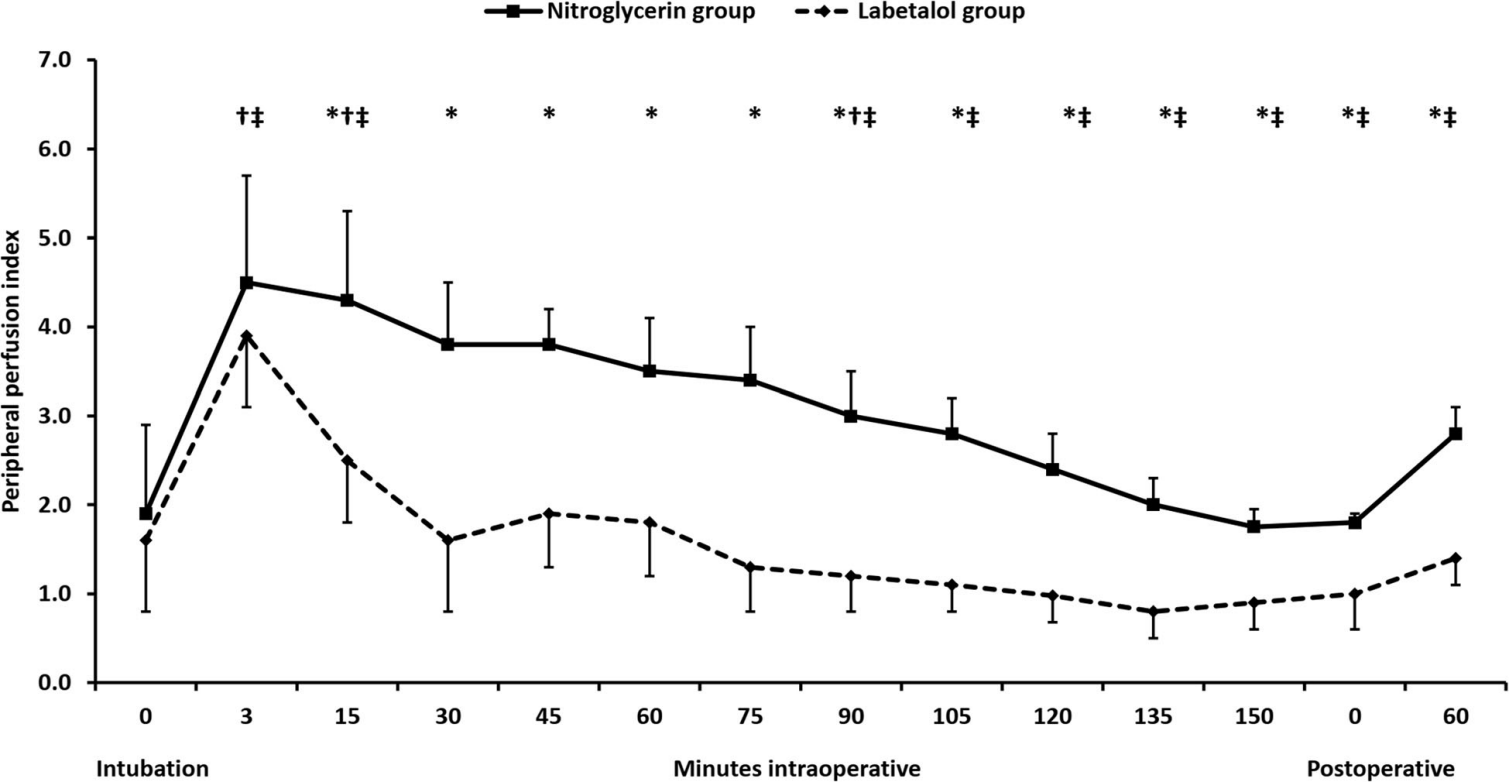
Peripheral perfusion index. Markers are means, error bars are standard deviations. * denotes significance between both groups., † denotes significance compared to the baseline reading within nitroglycerin group, ‡ denotes significance compared to the baseline reading within labetalol group

Mean arterial pressure was comparable between the study groups at most of the time points (Fig. 2). Heart rate was lower in the labetalol group compared to the nitroglycerin group starting from 90 min until 135 min after induction of anesthesia (Fig. 3). The PPI was higher in the nitroglycerin group than in the labetalol group at nearly all records (Fig. 4). Postoperative serum lactate levels were modestly lower in the nitroglycerin group than in the labetalol group (Table 1).

SFS was modestly lower in the first 20 min after drug infusion in the labetalol group compared with the nitroglycerin group. Subsequent SFS values in both groups were acceptable (< 3) until the conclusion of surgery (Table 2). Intraoperative blood loss was comparable in both groups (Table 1).

**Table 2:** Outcomes measurements

	Nitroglycerin group (*n* = 20)	Labetalol group (*n* = 20)	*P* value
Lactate pre-induction (mmol/L)	0.4 ± 0.1	0.5 ± 0.2	0.261
Lactate post-operative (mmol/L)	1.3 ± 0.2	1.7 ± 0.4^a^	0.001
Intra-operative Blood loss (ml)	64 ± 13.3	57 ± 9.3	0.062
**Surgical field score:**			
10 min	3(2.5–3)	2(2–3) ^a^	0.009
20 min	2(2–2.5)	1.5(1–2) ^a^	0.001
30 min	2(1–2)	1(1–2) ^a^	0.018
40 min	1(1–1)	1(1–1)	0.89
50 min	1(1–1)	1(1–1)	0.97
60 min	1(1–1)	1(1–1)	0.79
70 min	1(1–1)	1(1–1)	0.99
80 min	1(1–1)	1(1–1)	1
90 min	1(1–1)	1(1–1)	1
100 min	1(1–1)	1(1–1)	1
110 min	1(1–1)	1(1–1)	1
120 min	1(1–1)	1(1–1)	1

Data are presented as mean ± SD or median (interquartile range). ^a^denotes statistical significance compared with nitroglycerin group; *P* < 0.05. Data were analyzed using the unpaired t test and the analysis of variance for repeated measures as appropriate

## Discussion

Results of our study revealed that nitroglycerin-induced deliberate hypotension was associated with more preserved PPI compared with labetalol-induced deliberate hypotension. Both study drugs provided acceptable surgical conditions; however, nitroglycerin infusion appeared to be safer in terms of peripheral tissue perfusion. We also found that using nitroglycerin for induction of controlled hypotension did not impair peripheral tissue perfusion.

There are two possible mechanisms that may account for the superiority of peripheral perfusion in nitroglycerin compared to labetalol. 1. Administration of vasodilator drugs improves microcirculatory blood flow by increasing precapillary inflow pressure [12, 13]. 2. Nitroglycerin is a vasodilator with specific effects on peripheral perfusion dynamics. It acts on the venous capacitance vessels, resulting in a decrease in venular outflow pressure, with further increase in pressure gradient in the microvascular bed. This may favor an increase in microvascular blood flow and microcirculatory recruitment [14]. On the other hand, Labetalol acts as an antagonist of α1 and β-adrenergic receptors at a ratio of 1:7 on intravenous administration; thus, it is considered to be a beta-blocker with some alpha-blocking activity [5, 8]. This explanation is supported by the positive effects of nitroglycerin infusion on microvascular flow in patients with septic shock [12, 13] and those with circulatory shock [14]. Furthermore, nitroglycerin-induced controlled hypotension was not associated with reduction in cerebral oxygen saturation or postoperative cognitive function [15].

We evaluated tissue perfusion using the oximetry-derived PPI. PPI is characterized as a non-invasive, real-time measure that increases during vasodilatation [16], and decreases during sympathetic stimulation and impaired peripheral perfusion [17, 18]. Moreover, parameters for evaluation of peripheral perfusion in the skin, including PPI, are increasingly being recommended as useful measures for body perfusion [19, 20]. PPI is used as an indicator of peripheral perfusion in intensive care units [20, 21] and operating theaters [4, 22]. A recent study reported that monitoring PPI reflects changes in systemic hemodynamics during general anesthesia in a setting of changing preload conditions [4]. We used the PPI for real-time monitoring of the peripheral perfusion while we measured the serum lactate at only two points because it is a slowly changing perfusion marker which is usually measured at 6-h intervals [3].

Nitroglycerin and labetalol were previously compared in achieving deliberate hypotension [6–9]. However, the results of previous studies did not totally favor one drug over the other. Our study revealed that SFS values were modestly lower in the labetalol group in the first 10 min; however, the median SFS in the nitroglycerin group at 20 min was 2 (IQR 2–2.5), which is sufficient for good field quality. The subsequent SFS values were comparable in both groups until the end of the operation. In our patients, we found that the nitroglycerin group exhibited a higher heart rate in some intraoperative periods, while heart rate was comparable between the groups in most of the other readings. El-Shamma et al. and Hadavi et al. reported that nitroglycerin infusion was associated with more prominent reflex tachycardia compared with labetalol [6, 8]. The difference between our results and those reported by El-Shamma et al. and Hadavi et al. may be due to the higher dose of nitroglycerin or the larger sample sizes of their studies [6, 8].

The “art” of hypotensive anesthesia is to achieve proper surgical conditions without suppressing perfusion of vital organs [1]. The risk for impairment of tissue perfusion has been the primary concern that limits the use of deliberate hypotension [23]. However, in our patients, we found that PPI did not decrease in the nitroglycerin group throughout the operation, despite the low blood pressure. Furthermore, serum lactate concentration was not significantly elevated in both groups. Therefore, we believe that deliberate hypotension should not be considered a state of circulatory shock. We assume that, under well-monitored peripheral perfusion, nitroglycerin could be a good choice to induce controlled hypotension if it is surgically indicated.

There were several limitations to our investigation, the first of which was that it was a single-center study. Second, we induced hypotension in only one type of surgery; thus, our findings with regard to surgical field may differ in other procedures. Third, our patients received the available drugs (i.e., isoflurane and fentanyl) in our hospital; the impact of study drugs in peripheral perfusion with other agents, such as sevoflurane and remifentanil, may require further research. Fourth, our findings are restricted to patients with no serious comorbidities. Finally, although we demonstrated that deliberate hypotension did not impair peripheral perfusion under the conditions of our study, more studies aimed at evaluating the effect of deliberate hypotension on the perfusion of vital organs are warranted.

## Conclusion

Nitroglycerin-induced deliberate hypotension was accompanied by higher PPI and lower serum lactate levels compared with labetalol-induced deliberate hypotension during sinus endoscopic surgery.

## Data Availability

Data are available from the authors upon reasonable request with permission from Cairo University.

## Abbreviations

FESS: Functional Endoscopic Sinus surgery
MAP: Mean Arterial Pressure
PPI: Peripheral Perfusion Index
SFS: Surgical Field Score
ANOVA: Analysis Of Variance
Ext: Extubation
PACU: post-anesthesia care unit
